# Relative bioavailability and comparative clinical efficacy of different ivermectin oral formulations in lambs

**DOI:** 10.1186/1746-6148-9-27

**Published:** 2013-02-11

**Authors:** Gonzalo Suárez, Luis Alvarez, Daniel Castells, Oscar Correa, Pietro Fagiolino, Carlos Lanusse

**Affiliations:** 1 Área Farmacología, Facultad de Veterinaria, Universidad de la República, Montevideo, Uruguay; 2 Laboratorio de Farmacología, Centro de Investigación Veterinaria de Tandil (CIVETAN), CONICET, Facultad de Ciencias Veterinarias, Universidad Nacional del Centro de la Provincia de Buenos Aires, Campus Universitario, (7000), Tandil, Argentina; 3 Área de Investigación del Secretariado Uruguayo de la Lana (SUL), Florida, Uruguay; 4 Laboratorio de Parasitología, Facultad de Veterinaria, Universidad de la República, Montevideo, Uruguay; 5 Departamento de Ciencias Farmacéuticas, Facultad de Química, Universidad de la República, Montevideo, Uruguay

## Abstract

**Background:**

Several oral ivermectin (IVM) formulations for use in sheep are available in the pharmaceutical veterinary market in different countries. All of them are indicated at the same dose rate to treat the gastrointestinal nematodes. However, there is a lack of information on the relative systemic exposure (plasma bioavailability) and clinical efficacy among oral formulations routinely used in sheep. The main goal of the work reported here was to perform a pharmaco-parasitological assessment of three different IVM oral formulations in lambs infected with multiple resistant gastrointestinal nematodes. The comparative drug systemic exposure (IVM plasma concentrations) and nematodicidal efficacies (clinical efficacy) in lambs were determined for a reference (RF) and two different test (T1, T2) IVM oral formulations. One hundred and fifty six (n= 156) healthy Corriedale lambs, naturally infected with multiple resistant gastrointestinal nematodes were allocated into four experimental groups (n=39). Animals in each group received treatment (200 μg/kg) with either the RF, one of the test IVM formulations or were kept as untreated control. Blood samples were collected over 15 days post-treatment (n=8). The IVM plasma concentrations were measured by high performance liquid chromatography with fluorescence detection. The faecal nematode egg count reduction test (FECRT) (n=39) and evaluation of the clinical efficacy were performed at day 14 post-treatment (n=6), where a predominance of IVM highly resistant nematodes was observed.

**Results and conclusions:**

Neither the overall kinetic behaviour nor the IVM systemic exposure differed among all the tested oral formulations. Equivalent efficacy results were obtained for the different preparations, with an evident therapeutic failure to control *Haemonchus* spp. and *Teladorsagia circumcincta*, which correlates with a high degree of nematode resistance to IVM.

## Background

Ivermectin (IVM), a member of the macrocyclic lactone antiparasitic drugs, exhibits a broad-spectrum of activity against gastrointestinal (GI) and lung nematodes [1] as well as against ectoparasites of clinical relevance in domestic animals [2,3]. In sheep and goats at the dose of 0.2 mg/kg, IVM efficacy claims included *Haemonchus* spp., *Teladorsagia circumcincta*, *Ostertagia trifurcata*, *Trichostrongylus* spp., *Nematodirus* spp., Cooperia spp., *Oesophagostomum* spp., *Chavertia ovina* and *Trichuris ovis*, among the most important nematodes [4]. Additionally, its extensive tissue distribution, low biotransformation and high plasma-gastrointestinal (GI) recycling assure its persistent activity. Consequently, IVM is the most widely used anthelmintic, and this extensive use has led to the selection and emergence of IVM-resistant nematode populations in several areas of the world [5]. This is particularly relevant taking into consideration the rapid spread of parasite resistance in sheep nematodes.

In Uruguay, the registration of a “new” anthelmintic formulation is only based in a field efficacy study; information related to the pharmacokinetic behaviour of the specific formulation is not required. Several oral IVM formulations for use in lambs are available in the pharmaceutical veterinary market in Uruguay. All of them are indicated at the same dose rate (0.2 mg/kg) to treat GI nematodes. However, the route of administration and the formulation type strongly affect IVM plasma pharmacokinetic behaviour [6,7]. Differential systemic exposures were observed in cattle after the subcutaneous administration of IVM formulated as different commercial formulations [7,8]. Furthermore, some drastic pharmacokinetic differences were observed among generic albendazole formulations available for use in sheep [9,10]. However, there is a lack of information on the relative bioavailability among oral IVM formulations in sheep. Additionally, the impact on clinical efficacy against either dose-limiting or resistant nematodes related to drug-absorption differences due to the type and/or quality of pharmaceutical preparation needs to be addressed.

Bioequivalence/Relative bioavailability of a given anthelmintic drug should serve as additional evidence of equivalence in activity [11]. The estimation of the relative bioavailability is useful to compare the extent of absorption of different drug formulations of the same active ingredient. Assuming that a relationship exists between plasma concentration of the active moiety and clinical efficacy, knowledge of the bioavailability and disposition kinetics of the active compound would be particularly useful in the development of dosage forms and for comparison of routes of administration/formulations [12].

The mail goals of the current work were: 1) to determine the comparative IVM systemic exposure (relative bioavailability) obtained after treatment with three different oral formulations available in Uruguay for use in sheep, and 2) to investigate the efficacy of the three preparations against IVM resistant nematode parasites.

## Results

Analytical procedures, including chemical extraction, derivatization and HPLC analysis of IVM in lamb plasma were appropriately validated. The linear regression lines for IVM in plasma in the range 0.1-2.0 ng/mL and 2.0-40 ng/mL showed correlation coefficients from 0.9994 to 0.9972 and the departure from linearity was not statistically significant. The intra and inter assay precision of the analytical procedures obtained after HPLC analysis of IVM on different working days showed CV 3.54% and 4.25%, respectively. The LOQ was established at 0.1 ng/mL.

**Figure 1:**
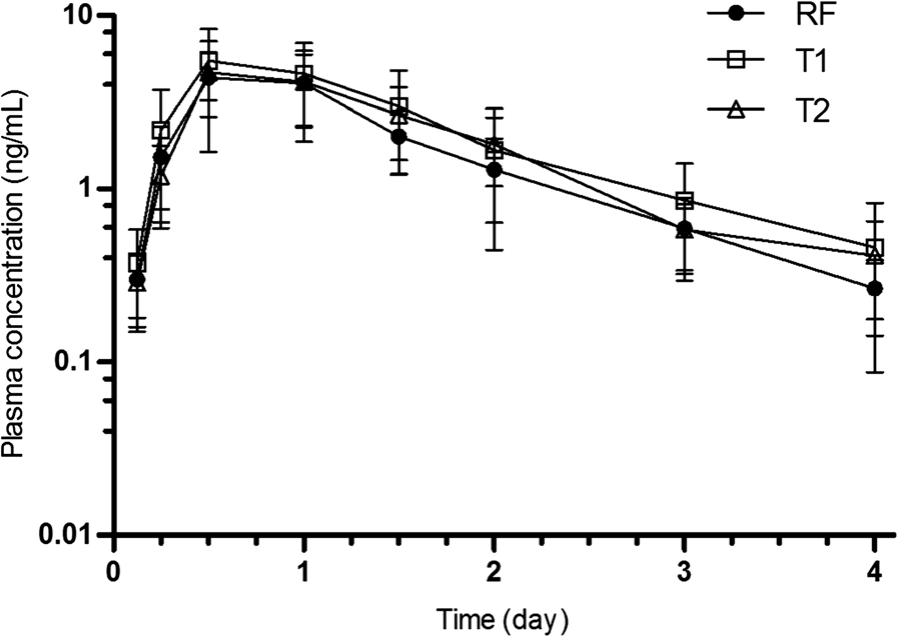
Mean (±SD) ivermectin plasma concentrations obtained after intraruminal administration of the Reference (RF), Test 1 (T1) and Test 2 (T2) formulations at200μg/kg in nematode infected lambs (n=8).

Figure 1 depicts the mean (±SD) IVM plasma concentration profiles obtained following the intraruminal (i.r.) administration of the RF (pioneer product) and each of the test generic (T1 and T2) IVM commercial formulation in parasitized lambs. Table 1 summarizes the main pharmacokinetic parameters obtained after the administration of IVM as the different assayed commercial formulations. IVM was first detected in plasma between 1 h and 6 days post-administration. The overall disposition kinetic of IVM was similar following treatment with each formulation. No statistical differences among formulations were observed in the different pharmacokinetic variables, including those related to IVM distribution (Vd_area_/F) and elimination (MRT, T_1/2el_, Cl_ƛ _/F) patterns (Table 1). IVM relative bioavailability was 132 and 117% for T1 and T2 formulation, respectively.

**Table 1:** Mean (±SD) ivermectin pharmacokinetic parameters obtained after the intraruminal administration of the Reference (RF), Test 1 (T1) and Test 2 (T2) formulations at 200 μg/kg in nematode infected lambs (n=8)

**Parameters**	**Reference**	**Test 1**	**Test 2**	**P value**
C_max_ (ng/mL)	5.14 ± 2.46	5.82 ± 2.53	4.96 ± 1.21	0.776
T_max_ (days)	0.81 ± 0.26	0.71 ± 0.27	0.60 ± 0.22	0.341
AUC_0-LOQ_ (ng.days/mL)	6.92 ± 3.26	9.18 ± 5.20	8.11 ± 3.27	0.568
AUC_0-∞_ (ng.days/mL)	7.20 ± 3.23	9.39 ± 5.20	8.35 ± 3.29	0.588
AUC_0-LOQ_/AUC_0-∞_	0.96 [3.9%]	0.98 [2.3%]	0.97 [2.9%]	-
MRT (days)	1.61 ± 0.24	1.60 ± 0.40	1.75 ± 0.39	0.724
T_1/2el_ (days)	1.07 ± 0.35	0.90 ± 0.21	1.12 ± 0.45	0.492
C_max_/AUC_0-LOQ_	0.74 ± 0.13	0.67 ± 0.22	0.64 ± 0.12	0.542
CL_ʎ _/F (L/days)	32.3 ± 12.2	25.6 ± 10.2	27.3 ± 10.9	0.505
Vd_area_/F (L/days)	48.5 ± 19.5	32.4 ± 13.5	40.5 ± 13.2	0.189

C_max_: peak plasma concentration; T_max_: time to peak plasma concentration; AUC_0-LOQ_: area under the concentration vs. time curve form 0 up to the limit of quantification; AUC0_-∞_: area under the concentration vs. time curve extrapolated to infinity; MRT: mean residence time; T_1/2el_: elimination half-life. CL_ƛ _/F: apparent total body clearance; Vd_area_/F: apparent volume of distribution (area method). Vd_area_ and CL_ƛ _ represent their true values divided by the systemic availability (F) of either drug. In values within a row no statistical differences were observed (P> 0.05). The percentage that AUC_0-LOQ_ differs from AUC_0-∞_ is reported in bracket [].

The mean (±SD) eggs per gram of faeces (epg) counts at 14 days after treatment for RF, T1, T2 and untreated control (n=39) were 5029 (±3673); 5100 (±3817); 5481 (±3831) and 5413 (±4349), respectively. *Haemonchus* spp. represented 99-100% of the total L_3_ recovered from fecal cultures in all group. The adult nematode counts and the efficacy results obtained for the different treatments are shown in Table 2. Upon necropsy, worms were recovered from all the IVM treated groups. The *Haemonchus* spp. genus resulted to be the most prevalent in the untreated control group. Besides, *T. circumcincta*, *Trichostrongylus* spp., *Nematodirus* spp., *Cooperia* spp., *Oesophagostomum* spp. and *Trichuris ovis* were recovered in a lower number.

**Table 2:** Mean number of worms (range) and efficacy (%) from necropsy performed 14 days after the intraruminal administration of the reference (RF) and each of test generic (Test 1 and Test 2) ivermectin formulations at 200 μg/kg in nematode infected lambs (n=6)

**Parasites**	**Reference**		**Test 1**		**Test 2**		**Untreated control**
	**Worm counts**	**Efficacy (%)**	**Worm counts**	**Efficacy (%)**	**Worm counts**	**Efficacy (%)**	**Worm counts**
**Abomasum**							
*Haemonchus* spp.	3210	**7**	3117	**8**	2514	**27**	3362
	(2190-4430)		(1920-3850)		(1591-3950)		(2680-4360)
*Teladorsagia circumcincta*	292	**39**	189	**57**	272	**44**	520
	(90-530)		(100-310)		(680-130)		(240-1460)
*Trichostrongylus axei*	73	**96**	47 *	**96**	140	**97**	375
	(0-220)		(0-160)		(0-440)		(80-1160)
**Small intestine**							
*Trichostrongylus columbriformis*	10 *	**100**	20	**94**	3 *	**100**	140
	(0-40)		(0-50)		(0-10)		(10-380)
*Cooperia* spp.	40	**80**	28	**38**	17	**61**	70
	(0-150)		(0-50)		(0-40)		(0-190)
*Nematodirus* spp.	25	**83**	38	**78**	5 *	**100**	278
	(0-60)		(0-90)		(0-30)		(0-980)
**Large intestine**							
*Oesophagostomun* spp.	0 *	**100**	0 *	**100**	0 *	**100**	16
	(0-0)		(0-1)		(0-0)		(2-19)
*Trichuris ovis*	0 *	**100**	0 *	**100**	0 *	**100**	5
	(0-0)		(0-0)		(0-0)		(2-10)

The percentage of efficacy was calculated using geometric mean as suggested by Wood et al. (1995).

* Nematode counts are statistically different (P< 0.05) compared to counts obtained in the untreated control group.

In this field study, the indirect efficacy estimated by means of the FECRT showed a low percentage of reduction for the RF (7.1%) as well as for the T1 (5.8%) and T2 (0%) formulation in comparison to the untreated control (P> 0.05). The efficacy of all the formulations demonstrated that *Haemonchus* spp. was basically refractory to the IVM treatment (<27%). The worm count data are in concordance with the FECRT and larval differentiation data in fecal cultures. Low efficacy (<80%) against *T. circumcincta*, *Cooperia* spp. and *Nematodirus* spp. was observed in the IVM treated groups. In contrast, IVM demonstrated to be highly efficacious against *Trichostrongylus* spp. in abomasum and small intestine. Due to variations in individual *Trichostrongylus* spp. counts, the observed differences did not reach statistical significance (P>0.05) compared to the untreated control in abomasums (RF and T2) and small intestine (T1). In all treated groups, no worms were recovered from the large intestine.

## Discussion

The macrocyclic lactones are the most widely used broad-spectrum antiparasitic drugs in veterinary medicine. Their notorious popularity is related to a high efficacy against ecto and endo parasites (nematodes), high potency, persistent activity and low toxicity. IVM, the first commercially available macrocyclic lactone endectocide, was introduced in the pharmaceutical market in the early ´ 80s. Since the IVM patent protection expire, several “similar” (generic) products entered the veterinary market worldwide. Uruguay was not an exception, according official data more than 60 different IVM formulations are currently registered for use in veterinary medicine, from which thirteen are solutions for oral administration to be used in sheep. The large number of available commercial formulations, situation that is reflected in many other countries around the world, faces the problem of a lack of information on their absorption patterns, which seems to be critical considering the possibility of differences on manufacturing processes and quality of components that may exist among formulations. These differences may substantially affect drug dissolution and its consequent GI absorption, which in turns could affect drug effectiveness. The comparison of the systemic drug exposure (measured as plasma concentration profiles) after treatment with different IVM generic formulation is an initial approach to check their pharmacotechnical quality, which has been shown to drastically affect the systemic availability of other active ingredients (i.e. albendazole) [10]. A RF and two generic IVM preparations were selected to be tested in the work reported here. The selection of the formulations did not respond to any particular interest to compare the quality among them. However, the comparison was done in order to simulate a real practical situation that could result useful to illustrate a market situation with a great impact on parasite control.

In order to assess the pharmacokinetic behaviour of different formulations, absorption related pharmacokinetic parameters must primarily be considered. The AUC, which reflects the extent to which the active drug is absorbed and is independent of the rate of the absorption process, and C_max_, which indicates the extent and the rate of drug absorption. Since differences in body condition, breed, gender, feeding, and parasitism substantially affect the plasma disposition kinetics of macrocyclic lactones (reviewed by [13]), the current study was conducted in lambs with similar characteristics, uniformly distributed among experimental groups. This is particularly important for studies conducted using a parallel design, since this experimental design has a lower power than the cross-over design for relative bioavailability [14]. However, the use of a parallel design can provide useful information on gross deficiencies in the absorption process of different anthelmintic formulations [10]. Similar (P> 0.05) IVM plasma AUC and Cmax (Table 1) were observed among formulations, suggesting a similar extent of absorption among the addressed reference and generic formulations. Tmax and Cmax/AUC did not show significant differences among the studied formulations, revealing a similar rate of the absorption process. Furthermore, our study showed similar values for other pharmacokinetic parameters (Table 1). Since a similar pharmacokinetic behavior was observed for IVM after the administration of the RF compared to both test formulations in animals grazed on pasture, it could be concluded that the assayed commercial preparations deliver IVM in an equivalent way which may indicated that manufacturing and overall pharmaceutical quality did not differ among them.

A lower IVM plasma drug exposure (expressed as Cmax and AUC) was observed in the current experiment, compared to that previously reported [15]. This may be related to differences in some experiment-related factors (parasitism, breed, body condition, feed, etc.) which have shown to affect the pharmacokinetic behaviour of IVM [13]. On the other hand and as it was previously reported, IVM plasma concentrations is higher after the SC compared to the IR administration [16,17]. Although similar concentration profiles were measured in the abomasal mucosa after treatment by both routes, markedly lower IVM concentrations were recovered in the abomasal contents after its SC injection. While the active secretion of IVM from the bloodstream to the abomasal lumen is of little relevance [18], the adsorption of IVM to ruminal particulate material may account for its low oral bioavailability which was estimated in about 25% [19].

High prevalence of anthelmintic resistance has now been reported in all parts of the world for GI helminth parasites, being nematodes of sheep and goats commonly involved [5,20,21]. In Uruguay, the development of anthelmintic resistance in sheep nematodes is not an exception. Resistance to IVM in sheep nematodes increased from 1.2% [22] to 65% [15] between 1996 and 2002. The trial described here demonstrated that current IVM resistance situation at the farm in which the study was conducted, is dramatically serious. The initially high IVM efficacy against GI nematodes in sheep has now drastically fell down, with almost a complete therapeutic failure to control some GI nematodes. Efficacies (evaluated by means the FECRT) as low as 7.1, 5.8 and 0% were observed for the RF, T1 and T2 IVM preparations under assay, respectively.

The identification of adult worms in the untreated lambs permitted to establish that the lambs were infected with *Haemonchus* spp., *T.circumcincta*, *Trichostrongylus* spp., *Nematodirus* spp., *Cooperia* spp., *Oesophagostomum* spp. and *T.ovis*. Nematode resistance in the current experiment was mainly related to *Haemonchus*spp., where all the IVM formulations failed to control this abomasal parasite. However, the clinical efficacy study also revealed a resistance-mediated failure to control *T. circumcincta*, where only efficacies ≤ 57% were observed. Resistance of *T. circumcincta* to IVM in Uruguay is reported here for the first time, which it may be useful as an indicator of the complexity of the resistance development phenomenon and its impact on livestock production.

All the tested IVM formulations also failed to control *Cooperia*spp*.* and *Nematodirus* spp. However, the low number of these parasites in the untreated control animals, limited the relevance of this finding. Contrarily, IVM demonstrated to maintain high efficacy against *Trichostrongylus* spp., *Oesophagostomum* spp. and *T.ovis*. Only *Haemonchus* spp. L_3_ larvae were recovered from the fecal cultures obtained from all the IVM treated groups. However, larvae obtained from fecal cultures are not necessarily related to parasites found at necropsies, since the high egg output observed in *Haemonchus spp*. may “mask” other nematodes.

Oppositely to what has been observed for other anthelmintics (such as the benzimidazole compounds), no significant differences on relative bioavailability/systemic exposure were observed among the tested IVM oral formulations in lambs. It is likely that any pharmaceutical/manufacturing change may more deeply affect the systemic availability of those compounds where GI absorption largely depends on the dissolution of low water soluble drug particles (suspension) in the abomasal lumen (i.e. albendazole), compared to the more lipophilic compounds such as IVM, but formulated as a mixed organic/aqueous solution. In spite of the fact that all the IVM formulations showed to reach an equivalent systemic exposure, all of them failed to control some common GI nematodes. The resistance status observed at the farm where the current trial was conducted is likely to be an indicator of the overall situation of the sheep flocks in Uruguay, and perhaps in many other regions of the world, where IVM completely failed to control *H.contortus*. This overall picture described in Uruguay, with resistance extended into other avermectin-type compounds, may be even worse if we consider that resistant *T. circumcincta* has been reported for the first time.

## Conclusions

Neither the overall kinetic behaviour nor the IVM systemic exposure differed among all the tested oral formulations. Equivalent efficacy results were obtained for the different preparations, with an evident therapeutic failure to control *Haemonchus* spp. and *T. circumcincta*, which correlates with a high degree of nematode resistance to IVM.

## Methods

### Chemicals

Standards of IVM and abamectin (ABA), used as internal standard, were obtained from Sigma Chemical Company (Saint Louis, MO, USA). Three oral IVM formulations approved and commercially available to use in sheep in the pharmaceutical market in Uruguay, were used in the current experiment. The comparison included: *Ivermectina 0.2 oral®* (IVM 0.2%, Rosenbusch, Uruguay); *Ivermic 0.2%®* (IVM 0.2%, Microsules, Uruguay) and *Ivomec® oral* (IVM 0.08%, Merial, The Netherlands). Ivomec® oral was considered the reference product (RF) as it was the pioneer first authorized product with a full dossier (NADA 131–392; approval date: July 26, 1988). The two IVM generic formulations were randomly designated as Test 1 (T1) and Test 2 (T2), respectively.

### Animals

The study was conducted in a farm (*Centro de Investigación y Experimentación “Dr. Alejandro Gallinal”, Florida, Uruguay*) where the failure of IVM to control GI nematodes had been previously demonstrated by the fecal egg counts reduction test (FECRT) [23]. One hundred and fifty six (n= 156) healthy male and female Corriedale lambs, not older than 1 year, weighing 29.5 ± 5.6 kg, body condition 3.1 ± 0.6, FAMACHA 1 [24] and naturally infected with GI nematodes, were involved in the trial. The criterion of inclusion for selection of the animals was based on worm egg per gram counts (epg) (>200 and < 8000 epg), body weight (≥ 20 and ≤ 45 kg), FAMACHA 1 and body condition (≥ 2 and ≤ 4) [25]. Throughout and 60 days before starting the experiment, animals grazed on a natural pasture and had free access to water. Animal procedures and management protocols were approved by the Ethics Committee according to the Animal Welfare Policy of the Faculty of Veterinary Medicine, Universidad de la República, Montevideo, Uruguay (http://www.fvet.edu.uy).

### Experimental design and treatments

On day −1, the experimental animals had an average of 2063 ± 1635 epg. The animals were ranked from lowest to highest epg counts. Based on increasing epg counts, replicates of 4 animals were formed. Within each replicate, animals were randomly assigned to treatment. The study was designed to have 39 animals per treatment group. One group of lambs was processed as the treated animals, but without drug treatment (untreated control). Animals in the other groups were treated with either the RF or each of the generic (T1 and T2) IVM formulations. All the IVM formulations were administered by the i.r. route at the dose rate of 0.2 mg/kg bodyweight. The i.r. route of administration was chosen in order to avoid leak/regurgitation of the administered dose and/or oesophageal grove closure, which commonly occurs after oral treatments affecting drug systemic availability [26]. Eight animals from each experimental group were randomly selected for the pharmacokinetic trial; being six of them used for the clinical efficacy trial. After selection, animals from the different groups involved in the pharmacokinetic and clinical efficacy trials have epg counts of 1489±252.

### Sampling

#### Pharmacokinetic trial

Heparinized blood samples (5 mL) were collected by jugular venipuncture prior to drug administration and at 1, 3, 6, 12, 18, 24, 36, 48 h, and 3, 6, 9, 12 and 15 days post-treatment. Blood samples were centrifuged at 3000 × g for 15 min and plasma was transferred to plastic tubes. All the plasma samples were stored at −20°C until analyzed by high performance liquid chromatography (HPLC).

#### Efficacy trial

Individual fecal samples were collected from the rectum of each animal (n= 39 each group) at 14 days post-treatment to assess the epg counts. Additionally, pooled samples were carried out in each experimental group for the coprocultures following the method described by Coles et al. [27]. Fourteen days after the treatment, six animals per experimental group were slaughtered for helminth recovery according of Veterinary Parasitology (WAAVP) guidelines [11]. The genera present in each GI compartment were identified and counted for each animal, separately, according the Ministry of Agriculture, Fisheries and Food [28], guidelines.

### IVM Analytical procedures

#### Sample clean-up and derivatization

The extraction of IVM, from spiked and experimental plasma samples was carried out following the well-established technique [29]; slightly modified by 6). Aliquots of plasma (1 mL) sample was fortified with 20 μL of ABA (20 μg/mL) (use as an internal standard) and acetonitrile (1 mL). Deionized water (0.250 mL) was added to each sample. The preparation was mixed using MultiTubevortexer (VWR Scientific Products, USA) for 20 min and the solvent-sample mixture was centrifuged at 2000 g during 10 min. The supernatant was manually transferred into a tube. The supernatant was applied to a conditioned disposable C18 column (RP-18, 100 mg, Strata®, Phenomenex, CA, USA), previously conditioned by passing 2 mL methanol and 2 mL deionized water. After washing with 1 mL of deionized water followed by 1 mL of water/methanol (4:1), the cartridges were dried for 5 min and the compounds were eluted with 1.5 mL of methanol and concentrated to dryness under a stream of nitrogen at 56°C in a water bath. The resuspension was carried out with 100 μL of a solution of N-methylimidazole (Sigma Chemical, St. Louis, MO, USA) in acetonitrile (1:1) [30]. Derivatization was initiated by adding 150 μL of trifluoroacetic anhydride (Sigma Chemical, St Louis, MO, USA) solution in acetonitrile (1:2). After completion of the reaction (<30 sec), an aliquot (100 μL) of this solution was injected directly into the chromatograph.

#### High performance liquid chromatography (HPLC) and validation

IVM concentrations were determined by HPLC using a Shimadzu 10A HPLC system with autosampler (Shimadzu Corporation, Kyoto, Japan). HPLC analysis was undertaken using a reverse phase C18 column (Phenomenex, 5 μm, 4.6 mm × 250 mm) and an acetic acid 0.2% in water⁄ methanol⁄ acetonitrile (3.8⁄ 40⁄ 56.2) mobile phase at a flow rate of 1.5 mL⁄ min at 30°C. IVM was detected using a fluorescence detector (Shimadzu, RF-10A Spectrofluorometric detector, Kyoto, Japan), readings at 365 nm (excitation wavelength) and 475 nm (emission wavelength). IVM concentrations were determined by the internal standard method using the Class LC 10 Software version 1.2 (Shimadzu Corporation, Kyoto, Japan) on an IBM compatible AT computer. The peak area ratios were considered to calculate the IVM concentrations in spiked (validation) and experimental plasma samples. There was no interference of endogenous compounds in the chromatographic determinations. The solvents (Baker, Phillipsburg, NJ, USA) used during the extraction and drug analysis were HPLC grade.

### Method validation

A complete validation of the analytical procedures used for extraction and quantification of IVM was performed before starting analysis of the experimental samples obtained during the pharmacokinetic trial. Calibration curves in the range between 0.1-2.0 ng/mL and 2.0-40 ng⁄ mL were plotted using the peak area ratios between analyte and the internal standard. Calibration curves were established using least squares linear regression analysis and correlation coefficients (r) and CV calculated. Linearity was established to determine the IVM concentrations/detector responses relationship. Percentages of IVM recovery from plasma were obtained in the range between 0.2 and 20 ng/mL. The inter-assay precision of the extraction and chromatography procedures was estimated by processing replicate aliquots (n = 6) of pooled sheep plasma samples containing known IVM concentrations (0.1-2.0 and 2.0-40 ng ⁄ mL) on different working days. The CV for recovery and inter-day precision of the method were calculated. The limit of detection (LOD) was estimated according to the following equation [24]: LOD= A/B + (SD * 3), where A is the baseline threshold at the retention time of each compound (n= 6) in spiked plasma samples, B the peak area of the internal standard (ABA), and SD the standard deviation obtained from A. The limit of quantification (LOQ) was defined as the lowest measured concentration with a CV <20% an accuracy of ±20% and an absolute recovery ≥70%. Concentration values below the LOQ were not considered for the kinetic analysis of experimental data.

### Pharmacokinetic analysis of the data

Non-compartmental pharmacokinetic calculations for the concentration versus time curves for IVM in plasma for each individual animal after the different treatments were conducted using the R software (version 2.14.0). The peak concentration (C_max_) and time to peak concentration (T_max_) were recorded directly from the measured concentration data. The elimination half-life (T_½el_) was calculated as ln 2⁄ λ_el_, where the terminal elimination rate constant (λ_el_), was calculated by performing regression analysis using data points belonging of the terminal phase concentration-time plot. The area under the plasma concentration-time curve from zero up to the limit of quantification (AUC_0-LOQ_) was calculated by means of the trapezoidal rule [31] and further extrapolated to infinity (AUC_0-∞_) by dividing the last experimental concentration by the terminal elimination rate constant (λ_el_). Statistical moment theory was applied to calculate the mean residence time (MRT) by using the formula MRT= AUMC⁄ AUC_0-LOQ_[32] where AUMC is the area under the curve of the product of time and the plasma drug concentration vs. time from zero to infinity [31], and AUC_0-LOQ_ is as defined above. C_max_/AUC_0-LOQ._ was calculated by dividing the C_max_ by AUC_0-LOQ_. The distribution and elimination were calculated as plasma clearance per fraction of the dose absorbed (CL/F) calculated using AUC_0-LOQ_ and apparent volume of distribution during the elimination phase per fraction of the dose absorbed (Vd_λel_/F). Relative bioavailability (F%) was measured by comparing the AUC_0-LOQ_ of the Test formulation with the AUC_0-LOQ_ of the RF, using the following equation [33]:

$$F\%={\mathrm{AUC}}_{\mathrm{test}}/{\mathrm{AUC}}_{\mathrm{RF}}*100$$

### Efficacy assessment

The FECRT were calculated according to the method described in the WAAVP recommendations for detection of anthelmintic resistance [27]. The percentage of efficacy (% E) of each anthelmintic treatment against a given parasite species was determined by the comparison of worm burdens in treated (groups RF, T1 and T2) versus untreated control animals using the following formula [11]:% E = (geometric mean of controls – geometric mean of treated/geometric mean of controls) × 100. The genera and species of the third stage larvae recovered from faecal pool cultures or adult nematodes recovered from parasitized lambs (Groups RF, T1, T2 and untreated control) were identified following the Ministry of Agriculture, Fisheries and Food [28] guidelines.

### Statistical analysis of the data

The pharmacokinetic parameters, concentration data, epg and nematode counts are reported as arithmetic mean ± SD. Parametric (ANOVA + Tuckey) or non parametric (Kruskal-Wallis) test were used for the statistical comparison of the pharmacokinetic and efficacy data obtained from the different experimental groups. The assumption that the data obtained after treatments have the same variance was assessed. Prior to analysis, the individual epg and nematode counts were transformed using (log_10+n_). A value of *P< 0.05* was considered statistically significant. The statistical analysis was performed using the R software, version 2.14.0 [34].

## Competing interests

The authors declare that they have no competing interests.

## Authors’ contributions

GS conceived the study and participate in the animal and analytical phase of the experiment and in writing the draft manuscript. DC and LA conceived the study, participated in its design and in the animal phase and revised the draft version of the manuscript. CL conceived the study, participated in its design and revised the draft version of the manuscript. OC participated in the parasitological analysis and revised the draft version of the manuscript.PF participated in the pharmacological analysis and revised the draft version of the manuscript. All authors have read and approved the final manuscript.
